# A Compend of Dental Pathology and Dental Medicine

**Published:** 1893-07

**Authors:** 


### Bibliography.

A Compend of Dental Pathology and Dental Medicine. By George W. Warren, D D.S. Second Edition, Illustrated. Philadelphia: P. Blakiston, Son & Co., 1893.

The fact that a second edition of this Compend has been called for in so short a period since the first issue is an evidence that it has filled a needed place in dental education.

The author has achieved a fair measure of success in condensing important pathological conditions into small paragraphs,—more so, perhaps, than is usual in this character of books. Nothing so much indicates the growing tendency towards abbreviation and reducing the time given to study as these Compends. If properly used as aids to more thorough work they have a special value, but if depended on entirely they become misleading.

The criticism can fairly be made against this Compend that it devotes too much space to fractures and dislocations and too little to the every-day pathological conditions.

The portion devoted to materia medica is well prepared, and while not as full as it might be made, will be found an excellent hand-book for the student.

